# CARD14 Signalling Ensures Cell Survival and Cancer Associated Gene Expression in Prostate Cancer Cells

**DOI:** 10.3390/biomedicines10082008

**Published:** 2022-08-18

**Authors:** Domien Vanneste, Jens Staal, Mira Haegman, Yasmine Driege, Marieke Carels, Elien Van Nuffel, Pieter De Bleser, Yvan Saeys, Rudi Beyaert, Inna S. Afonina

**Affiliations:** 1 Unit of Molecular Signal Transduction in Inflammation, VIB-UGent Center for Inflammation Research, 9000 Ghent, Belgium; 2 Department of Biomedical Molecular Biology, Ghent University, 9000 Ghent, Belgium; 3 Unit of Data Mining and Modeling for Biomedicine, VIB-UGent Center for Inflammation Research, 9000 Ghent, Belgium; 4 Department of Applied Mathematics, Computer Science and Statistics, Ghent University, 9000 Ghent, Belgium

**Keywords:** CARD14, MALT1, protease, prostate cancer, tumour cell survival

## Abstract

Prostate cancer (PCa) is one of the most common cancer types in men and represents an increasing global problem due to the modern Western lifestyle. The signalling adapter protein CARD14 is specifically expressed in epithelial cells, where it has been shown to mediate NF-κB signalling, but a role for CARD14 in carcinoma has not yet been described. By analysing existing cancer databases, we found that CARD14 overexpression strongly correlates with aggressive PCa in human patients. Moreover, we showed that CARD14 is overexpressed in the LNCaP PCa cell line and that knockdown of CARD14 severely reduces LNCaP cell survival. Similarly, knockdown of BCL10 and MALT1, which are known to form a signalling complex with CARD14, also induced LNCaP cell death. MALT1 is a paracaspase that mediates downstream signalling by acting as a scaffold, as well as a protease. Recent studies have already indicated a role for the scaffold function of MALT1 in PCa cell growth. Here, we also demonstrated constitutive MALT1 proteolytic activity in several PCa cell lines, leading to cleavage of A20 and CYLD. Inhibition of MALT1 protease activity did not affect PCa cell survival nor activation of NF-κB and JNK signalling, but reduced expression of cancer-associated genes, including the cytokine IL-6. Taken together, our results revealed a novel role for CARD14-induced signalling in regulating PCa cell survival and gene expression. The epithelial cell type-specific expression of CARD14 may offer novel opportunities for more specific therapeutic targeting approaches in PCa.

## 1. Introduction

Most cancers arise in epithelial cells, manifesting as carcinomas in organs such as the lung, skin, breast, colon, and prostate. Prostate cancer (PCa) is one of the leading causes of cancer-related death in men worldwide, despite the fact that the overall survival rate after PCa diagnosis is relatively high [1,2], indicating the need for better treatment options. In the Western countries, PCa rates are particularly increased due to the wide occurrence of obesity, metabolic syndrome, and associated comorbidities that elevate inflammation, which has been linked to PCa susceptibility [3]. Inflammatory mediators, in turn, stimulate malignant proliferation of epithelial cells via activation of various signalling pathways, including NF-κB, ERK, or Akt [4,5]. Therefore, epithelial cell-specific signalling molecules are of particular interest for the development of new anticancer drugs.

CARD14 is an intracellular signaling protein whose activation nucleates the formation of an oligomeric CARD14-BCL10-MALT1 (CBM) complex leading to the activation of NF-κB and the MAP kinases p38, JNK, and ERK [6,7]. It is predominantly expressed in epithelial cells of placenta, skin, and mucosal tissues, although some isoforms were also shown to be expressed in hematopoietic cell lines [8]. CARD14 shares a similar structure and function with CARD11 and CARD10, with CARD11 being predominantly expressed in hematopoietic cells, while CARD10 is more broadly expressed [9]. In contrast to CARD10 and CARD11, CARD14-activating stimuli are still largely unclear; thus, CARD14 activation has mainly been studied in the context of CARD14 overexpression or by the use of specific CARD14 gain-of-function mutants identified from psoriasis patients [8,10]. In addition to initiating the formation of a signalling hub that enables docking and activation of downstream signalling mediators, CARD14 overexpression activates the proteolytic activity of MALT1 that is required for CARD14-induced inflammatory responses [6]. Activated MALT1 cleaves several substrates, including the deubiquitinases CYLD and A20 [11,12]. Interestingly, CYLD deficiency has been shown to promote PCa cell proliferation, cell survival, and tumorigenesis, and CYLD abundance is reduced in advanced-stage PCa samples, suggesting that CYLD may play a tumour-suppressive role in PCa [13].

MALT1 is frequently activated in multiple lymphoid (such as activated B cell-like diffuse large B cell lymphoma (ABC-DLBCL)) and non-lymphoid cancers (such as lung and breast carcinoma) [14,15,16,17,18]. Recently, several reports have also described a role for MALT1 in PCa [19,20]. MALT1 knockdown reduces proliferation, invasion, and migration in PCa cell lines, as well as attenuates xenograft tumour establishment in nude mice [19,20]. Although CARD11 and CARD10 have been previously shown to contribute to lymphoid malignancies and epithelial carcinomas, respectively [21,22,23,24], CARD14 signalling has so far been mainly studied in keratinocytes in the context of psoriasis [6]. However, elevated CARD14 expression is also observed in human epithelial cancers [25]. Furthermore, potential gain-of-function mutations in CARD14 have been identified in lethal castration-resistant PCa, suggesting that CARD14-mediated signalling may be involved in tumorigenesis [26]. In this study, we reported a novel role for CARD14 in regulating PCa cell survival and gene expression.

## 2. Materials and Methods

### 2.1. Antibodies and Reagents

The following antibodies were used: anti-MALT1 polyclonal antibody (ab33921, Abcam, Amsterdam, Netherlands), anti-CARD14 polyclonal antibody (10400-1-AP, ProteinTech, Manchester, United Kingdom), anti-BCL10 monoclonal antibody (sc-5273, Santa-Cruz, Heidelberg, Germany), anti-c-Jun monoclonal antibody (sc-74543, Santa Cruz, Heidelberg, Germany), anti-phospho-c-Jun (Ser63) polyclonal antibody (9261, Cell Signaling Technology, MA, USA), anti-IκBα polyclonal antibody (sc-371, Santa Cruz, Heidelberg, Germany), anti-phospho-IκBα (Ser32/36) monoclonal antibody (9246, Cell Signaling Technology, MA, USA), anti-Akt polyclonal antibody (9272, Cell Signalling, MA, USA), anti-phospho-Akt (Ser473) polyclonal antibody (9271, Cell Signalling, MA, USA), anti-CYLD monoclonal antibody (sc-74435, Santa Cruz, Heidelberg, Germany), anti-A20 monoclonal antibody (sc-166692, Santa Cruz, Heidelberg, Germany), and anti-actin monoclonal antibody (MP6472J, MP Biomedicals, Eschwege, Germany). HRP-conjugated goat anti-mouse IgG cross-adsorbed secondary antibody (31432) or mouse anti-rabbit IgG cross-adsorbed secondary antibody (31464) were from ThermoFisher Scientific (Merelbeke, Belgium).

A broad spectrum protein kinase C (PKC) inhibitor Gö6983 (2285) was purchased from Bio-Techne (Abingdon, United Kingdom), and MALT1 inhibitor (MLT-827, [27] was generously provided by Galapagos n.v., Mechelen, Belgium). MALT1 protease activity was followed by immunoblotting to detect cleavage of MALT1 substrates CYLD and A20.

### 2.2. Human PCa Data Sets

The Cancer Genome Atlas (TCGA) prostate adenocarcinoma (PRAD) gene expression data (RNA sequencing raw counts, z-score and RSEM) was downloaded from the Firehose website [28] Corresponding clinical data of patients were retrieved from the TCGA data portal [29]. Additional gene expression data (TCGA PRAD RSEM and MSKCC PRAD z-scores) and clinical data were curated from the cBioportal website [30].

### 2.3. Proliferation Score

The Proliferation Score or meta-PCNA index was calculated as previously described [31]. In brief, the meta-PCNA index of each sample was computed by taking the median expression of predefined genes that were found to be the top 1% most positively correlated with *PCNA* expression.

### 2.4. Gene Ontology (GO) Enrichment Analysis

We calculated the correlation between *CARD14* and all the genes in the TCGA PRAD data set and selected the significant positively correlated genes (*p* < 0.05 and *r* > 0). This list of genes was queried for the enrichment of Biological Processes on the “PANTHER” website [32].

### 2.5. Gene Set Enrichment Analysis (GSEA)

The GSEA 4.1.3 software was downloaded from Broad Institute [33]. GSEA on TCGA PRAD gene expression data was used to discover hallmark gene sets that are significantly enriched with *CARD14* expression. *CARD14* was used as a continuous phenotype label to rank patients from high to low *CARD14* expression. The enrichment of gene sets was tested with 1000 permutations. Gene sets with a positive enrichment score were enriched in patients with high *CARD14* expression, while gene sets with a negative enrichment score were enriched in patients with low *CARD14* expression.

### 2.6. DNA Damage Response (DDR) Pathway Activity

DDR gene list was assembled from an online catalogue of DDR genes from recently published resources [34]. This gene list was subdivided into eight major DDR pathways: the Fanconi anemia pathway, non-homologous DNA end joining, nucleotide excision repair, base excision repair, translesion DNA synthesis, damage sensor, homology-dependent recombination, mismatch repair, and direct damage reversal/repair. The complete gene list is contained in Table S1. DDR pathway activity was calculated by taking the mean expression of all the genes in that pathway.

### 2.7. Cells, siRNA-Mediated Knockdown and Cell Death Assays

Human prostate cancer cell lines LNCaP (ATCC CRL-1740) and PC3 (ATCC CRL-1435) were cultured in RPMI 1640 medium, supplemented with 10% fetal calf serum and 3 mM or 2 mM L-glutamine, respectively. Du145 (ATCC HTB-81) were cultured in Dulbecco’s modified Eagle’s medium, supplemented with 10% fetal calf serum and 2 mM L-glutamine. Immortalised normal adult prostatic epithelial cells PNT1A (ECACC 95012614) were cultured in RPMI 1640 medium, supplemented with 10% fetal calf serum, 2 mM L-glutamine and β-mercaptoethanol. INTERFERin^®^ (POL-409-10, Polyplus Transfection, Illkirch, France) was used for reverse transfection of 2 × 10^5^ cells with 20 nM of siRNA. SMARTpool ON-TARGET plus non-targeting pool control (D-001810-10), CARD14- (L-004397-00), BCL10- (L-004381-00) or siGENOME MALT1-specific (M-005936-02) siRNA were purchased from Dharmacon (Horizon Discovery Biosciences, Cambridge, United Kingdom). 24 h later SYTOX Green nucleic acid stain (S-7020, ThermoFisher Scientific, Merelbeke, Belgium) was added to final concentration of 1 µM and cells were incubated in a CO2 and temperature-controlled environment that allowed measurement of fluorescent signals over a time span of 72 h, as previously described [35]. The number of dead cells was quantified in function of time using the Incucyte Zoom Live-Cell Analysis System (Essenbio, Newark, UK). Values were normalised against a control well that was seeded with the same number of cells as in the experimental well and imaged in parallel following treatment with Triton X-100 to fully permeabilise the cells. The maximum number of positive nuclei in these control wells was considered 100% and used to normalise values of fluorescent cells in experimental wells. Gene knockdown was verified by immunoblotting 48–72 h later.

### 2.8. PC3 Genome Editing

Plasmids used in this study were deposited in the BCCM/GeneCorner plasmid collection (Ghent, Belgium) along with detailed descriptions of cloning strategy and plasmid sequence. Large-deletion MALT1ΔEx1-9 (clone #M24) PC3 cell line was generated via CRISPR/Cas9 genome editing using GFP-tagged Cas9 constructs ([36], pSpCas9(BB)-2A-GFP (PX458) was a gift from Feng Zhang (Addgene plasmid # 48138)). MALT1 was knocked-out by cotransfecting two Cas9:guide constructs targeting exon 1 (LMBP: 10533) and exon 9 (LMBP: 10401). Two days after transfection, single-cell sorting for GFP-positive (=successfully transfected) cells was performed on an ARIA II (BD Biosciences) fluorescence-activated cell sorter machine. Individual clones were evaluated for KO by PCR and functional assays. For reconstitution with MALT1, PC3 *MALT1* KO cells were stably transduced with the following lentiviral constructs: wild type MALT1 (pLVX-EF1α-hMALT1-IRES-ZsGreen1, LMBP: 08927), catalytically inactive MALT1 (pLVX-EF1α-hMALT1-C464A-IRES-ZsGreen1, LMBP: 08928) or parent vector (pLVX-EF1α-IRES-ZsGreen1, LMBP: 08652). Successfully transduced cells (GFP-positive) were sorted on an ARIA II fluorescence-activated cell sorter machine.

### 2.9. Multiplex Antibody Array Analysis of Protein Expression

7 × 10^6^ PC3 MALT1 KO or reconstituted cells were stimulated with 10 µg/mL Brefeldin A for 6 h. Cells were washed in PBS and lysed in R&D Lysis Buffer 17 (1% Igepal CA-630, 20 mM Tris-HCl (pH 8.0), 137 mM NaCl, 2 mM EDTA, 200 mM Sodium orthovanadate, 5 mM NaF). Protein concentration was measured using Bio-Rad protein assay dye reagent (5000006, Bio-Rad Laboratories, Nazareth, Belgium) and equal amounts of total lysate were analysed with Proteome Profiler Human XL Oncology Array (ARY026; R&D, Minneapolis, MN, USA) according to the manufacturer’s instructions. Membranes were exposed to X-ray film, after which profiles of mean spot pixel density were quantified using image analysis software.

### 2.10. IL-6 ELISA

For analysis of IL-6 secretion, LNCaP, Du145, and PC3 cells were seeded at 20,000–25,000 cells in 24-well plates and treated with 2 µM MALT1 inhibitor (MLT-827). Media was refreshed after 48 h with or without inhibitor and IL-6 was measured in cell supernatants after additional 48 h using human IL-6 ELISA (88-7066-88, ThermoFisher Scientific, Merelbeke, Belgium) according to the manufacturer’s instructions.

## 3. Results

### 3.1. CARD14 Is Associated with Aggressive PCa in Human Patients

**Figure 1:**
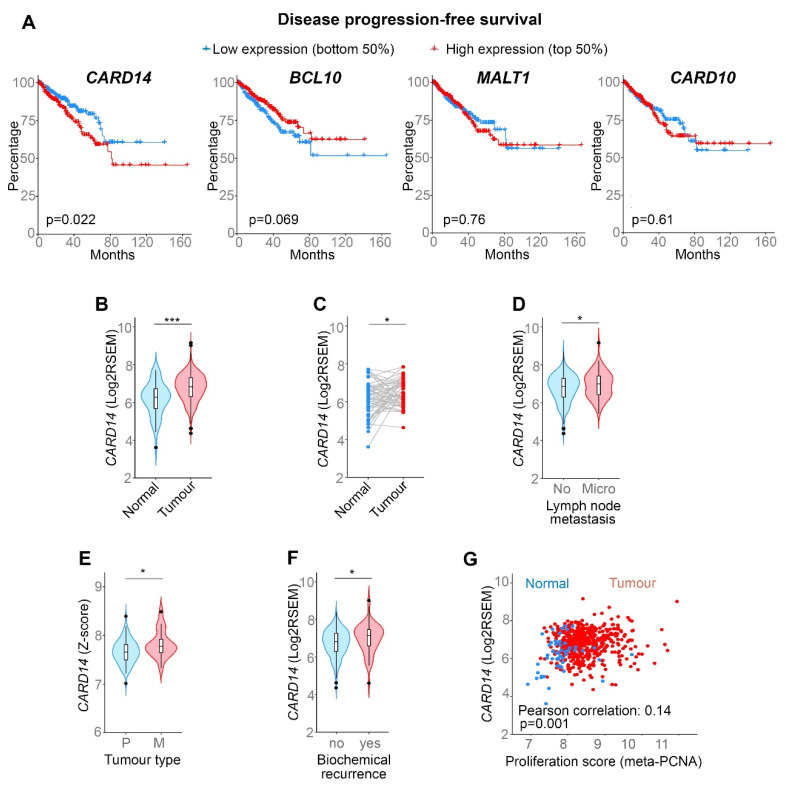
High *CARD14* expression correlates with aggressive cancer in human PCa patients. (**A**) Kaplan–Meier curve of the probability of disease-free survival for PCa patients with low or high *CARD14*, *CARD10*, *MALT1*, or *BCL10* RNA level, based on the TCGA RNAseq dataset. *p* value generated by the Log-rank test is indicated. (**B**,**C**) *CARD14* expression in normal and tumour (paired (**C**)) PCa tissue, based on TCGA data. (**D**–**F**) *CARD14* expression as a function of lymph node metastasis (**D**) (no: no presence of metastasis in lymph nodes; micro: presence of micrometastasis in lymph nodes); tumour metastasis (**E**) (P: primary tumour, M: metastatic tumour) or biochemical recurrence (**F**) for PCa, based on TCGA data (**D**,**F**) or MSKCC data (**E**). *p* value generated by the Welsh *t*-test is indicated as * *p* < 0.05, *** *p* < 0.001. (**G**) *CARD14* mRNA expression as a function of proliferation score.

To investigate whether CBM signalling may play a role in PCa, we initially analysed whether expression of CBM components correlates with survival of patients with PCa. To this end, we explored The Cancer Genome Atlas (TCGA) database and analysed Disease/progression-free survival of PCa patients expressing high or low levels of *CARD14*, *BCL10*, and *MALT1*. Because the CARD14-related protein CARD10 was previously reported to contribute to other epithelial carcinomas [21,22,23,24], we also analysed its expression in PCa. Patients with high *CARD14* expression were found to have a significantly lower Disease/progression-free survival compared with patients with low *CARD14* expression (Figure 1A). In comparison, expression of *MALT1*, *BCL10*, and *CARD10* did not correlate with disease progression (Figure 1A). In addition, expression of *CARD14* is higher in tumour tissue as opposed to (paired) healthy tissue (Figure 1B,C). Overexpression of *CARD14* was also significantly associated with lymph node metastasis (Figure 1D), while *CARD14* expression was found to be higher in metastatic PCa tumours compared with primary PCa tumours (Figure 1E). In agreement with this, TCGA analysis revealed that *CARD14* levels also correlate with cancer recurrence in PCa patients (Figure 1F). Finally, high *CARD14* expression was also found to correlate with higher proliferation in tumour tissue (Figure 1G). Together, these data show that high *CARD14* expression is associated with aggressive PCa in human patients.

### 3.2. CARD14 Expression Is Essential for PCa Cell Survival

**Figure 2:**
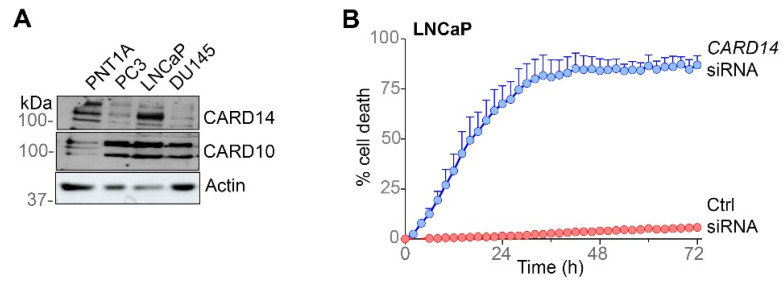
*CARD14* is essential for PCa cell survival. (**A**) *CARD10* and *CARD14* expression profile in PCa cell lines analysed by western blot. Actin expression was used as a loading control. (**B**) Cell death in function of time upon knock-down of *CARD14* in LNCaP cells. The number of dead cells was quantified using SYTOX green staining and the Incucyte Live-Cell Analysis System. Results are representative of three independent experiments. Error bars represent means ± SEs.

To further verify the in silico findings, we analysed expression of CARD14 and CARD10 proteins in three different PCa cell lines. While CARD10 was similarly expressed in LNCaP, PC3, and Du145 cells, CARD14 expression was much higher in LNCaP compared with Du145 and PC3 (Figure 2A). As Du145 and PC3 are known to be more aggressive than LNCaP, the CARD14 expression data in cell lines do not directly correlate with the above mentioned human PCa patient data, showing that higher *CARD14* expression correlates with aggressive cancer (Figure 1). The more aggressive nature of Du145 and PC3 most likely reflects other important factors such as their androgen-independence. To better understand the specific role of high CARD14 expression, we next silenced *CARD14* expression in LNCaP cells using siRNA and monitored cell survival over a 3-day period by analysing the uptake of the fluorescent dye SYTOX green, which can only enter dead cells with a permeabilised cell membrane. Survival of LNCaP cells was severely reduced in the absence of CARD14 (Figure 2B). These results suggest that CARD14-induced signalling contributes to PCa cell survival and tumorigenesis.

### 3.3. CARD14 Expression Correlates with DNA Repair Gene Enrichment

**Figure 3:**
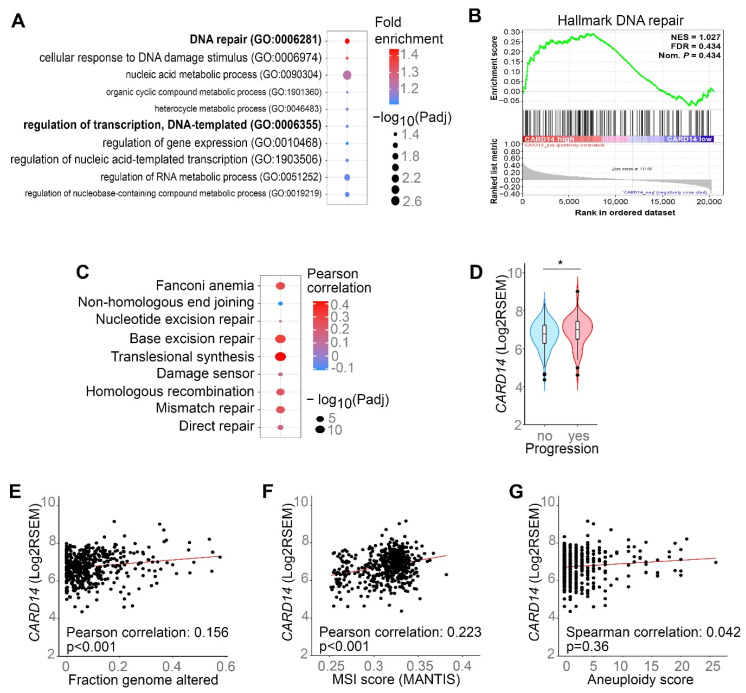
*CARD14* expression correlates with DNA repair gene enrichment. (**A**) Gene ontology analysis for enrichment of biological processes on all genes whose expression showed a significant correlation with *CARD14* expression based on gene expression (RSEM) in TCGA prostate adenocarcinoma (PRAD) patients. (**B**) Gene set enrichment analysis for enrichment of the Hallmark DNA repair gene set in genes ranked by positive correlation with *CARD14* expression in TCGA PRAD patients (NES: Normalised Enrichment Score, FDR: False Discovery Rate). (**C**) Pearson correlation analysis between DNA damage response (DDR) pathway activity and *CARD14* expression in TCGA PRAD patients. (**D**–**G**) *CARD14* mRNA expression as a function of disease progression after radiotherapy (**D**), fraction genome altered (**E**), microsatellite instability (**F**) and aneuploidy score (**G**) in PCa patients, based on TCGA data. *p* value in (**D**) was generated by Welch *t*-test, * *p* < 0.05.

To gain further insights into the biological processes regulated by *CARD14* signalling in PCa, we performed gene ontology (GO) analysis on genes that were significantly correlated with *CARD14* expression in the TCGA PRAD gene expression data. This analysis indicated that DNA repair genes were most enriched in PCa patients with high *CARD14* expression (Figure 3A). However, although additional gene set enrichment analysis (GSEA) also showed that DNA repair genes are enriched in PCa patients with high *CARD14* expression, the difference was not found to be statistically significant (Figure 3B). Therefore, we further investigated the relationship between *CARD14* expression and specific DNA damage response (DDR) pathways in more detail using the TCGA PRAD gene expression data. Several DDR pathways including Fanconi anemia, base excision repair, translesional synthesis, damage sensor, homologous recombination, mismatch repair, and direct repair were found to be positively correlated with *CARD14* expression (Figure 3C). While the biological role of DDR pathway activation is to suppress tumorigenesis by maintaining genome stability, high expression of DNA repair pathway machinery genes is known to contribute to tumour resistance to radiotherapy [37]. In agreement with this, we observed that in PCa patients who received radiotherapy, *CARD14* expression was higher in patients with disease progression when compared with patients who do not show disease progression (Figure 3D).

It has been recently reported that expression of two hyperactivating CARD14 mutants identified in Pityriasis rubra pilaris patients did not induce DNA damage or increase rates of homologous recombination upon expression in U2OS human bone osteosarcoma epithelial cells, but increased the incidence of double strand breaks (DSB) during prolonged replication stress and promoted break-induced replication [38]. The latter pathway repairs one-ended DSB and is associated with elevated levels of chromosomal rearrangements known to contribute to cancer development [39]. We also found that high *CARD14* expression in PCa patients somewhat correlates with a higher fraction genome altered and microsatellite instability, but not with aneuploidy score (Figure 3E–G). However, because the correlation is small (less than 0.25) albeit statistically significant (Figure 3E,F), the biological relevance of these data remains uncertain. Additional biological assays will therefore be needed in the future to support a possible link with genome instability.

### 3.4. PCa Cells Show Constitutive MALT1 Proteolytic Activity, but PCa Cell Survival Only Requires MALT1 Scaffold Activity

**Figure 4:**
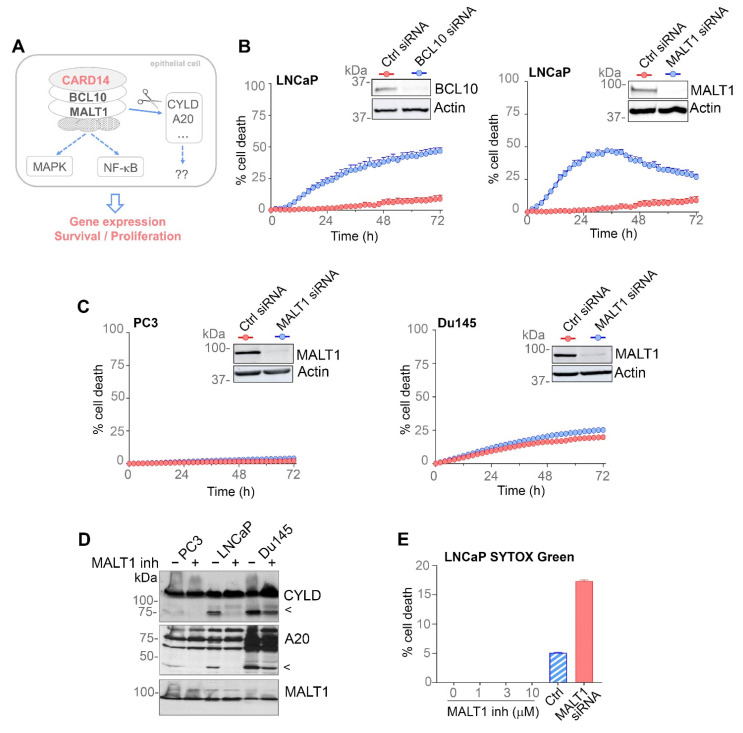
MALT1 protease activity is constitutively active in PCa cells, but PCa cell survival only requires MALT1 scaffold activity and not its catalytic activity. (**A**) Schematic representation of CARD14 signalling in epithelial cells. Activation of CARD14 by a yet to be defined upstream stimulus or a gain-of-function mutation triggers formation of a CARD14–BCL10–MALT1 complex leading to NF-κB- and MAPK-mediated gene expression. Additionally, CBM formation unlocks the ability of MALT1 to cleave several substrates that further regulate downstream events. (**B**,**C**) Cell death in function of time upon knock-down of *MALT1* or *BCL10* in LNCaP cells (**B**), PC3 and DU145 cells (**C**), as indicated. The number of dead cells was quantified using SYTOX green staining and the Incucyte Live-Cell Analysis System. (**D**) The indicated PCa cell lines were left untreated (−) or treated (+) with MALT1 inhibitor (1 µM) for 48 h. CYLD and A20 cleavage (indicated with an arrow head), as well as MALT1 expression was visualised by western blot of the corresponding cell extracts. (**E**) Cell death in function of time upon MALT1 inhibition in LNCaP cells, analysed as in (**B**). Results are representative of three independent experiments. Error bars (**B**,**C**,**E**) represent means ± SEs.

Activation of CARD14 nucleates the formation of a CBM (CARD14::BCL10::MALT1) signalling complex that is essential for propagation of downstream signalling (Figure 4A) [6,7]. Importantly, akin to *CARD14* silencing, *BCL10* and *MALT1* knockdown also reduced survival of LNCaP cells (Figure 4B). In contrast, *MALT1* knockdown had no effect on PC3 and DU145 cell survival, presumably reflecting the more aggressive nature of these cells due to their androgen independence (Figure 4C). The essential role for CARD14, BCL10, and MALT1 in LNCaP cell survival indicates the importance of CARD14-induced CBM formation, which is in agreement with the recently reported role for MALT1 in regulating proliferation and migration/invasion of PCa cells [19,20].

In addition to its scaffold function, CBM formation is known to unlock proteolytic activity of MALT1, which leads to cleavage of several substrates to fine-tune downstream signalling and gene expression (Figure 4A) [40]. We therefore investigated MALT1 proteolytic activity at the molecular level by analysing cleavage of the known MALT1 substrates CYLD and A20 via immunoblotting. Both substrates were constitutively cleaved in LNCaP cells (Figure 4D). Interestingly, although *MALT1* knockdown in PC3 and DU145 cells had no effect on cell survival (Figure 4C), DU145 and, to a lesser extent, PC3 cells also showed constitutive MALT1 proteolytic activity (Figure 4D). Since DU145 and PC3 show almost no CARD14 expression, activation of MALT1 proteolytic activity in these cells is possibly mediated by CARD10, which is strongly expressed in these cell lines (Figure 2A). Constitutive cleavage of MALT1 substrates in PCa cells was inhibited upon treatment with the selective small molecule MALT1 protease inhibitor MLT-827 (Figure 4D). However, in contrast to the strong effect of MALT1 silencing on LNCaP cell survival (Figure 4B), pharmacological inhibition of MALT1 catalytic activity with MLT-827 treatment had no effect (Figure 4E), suggesting that the scaffold function of MALT1 plays a primary role in regulating survival of LNCaP cells.

### 3.5. Proteolytic Activity of MALT1 Does Not Contribute to Constitutive NF-κB/JNK/Akt Signalling in PCa Cells, but Promotes Cancer-Associated Gene Expression

**Figure 5:**
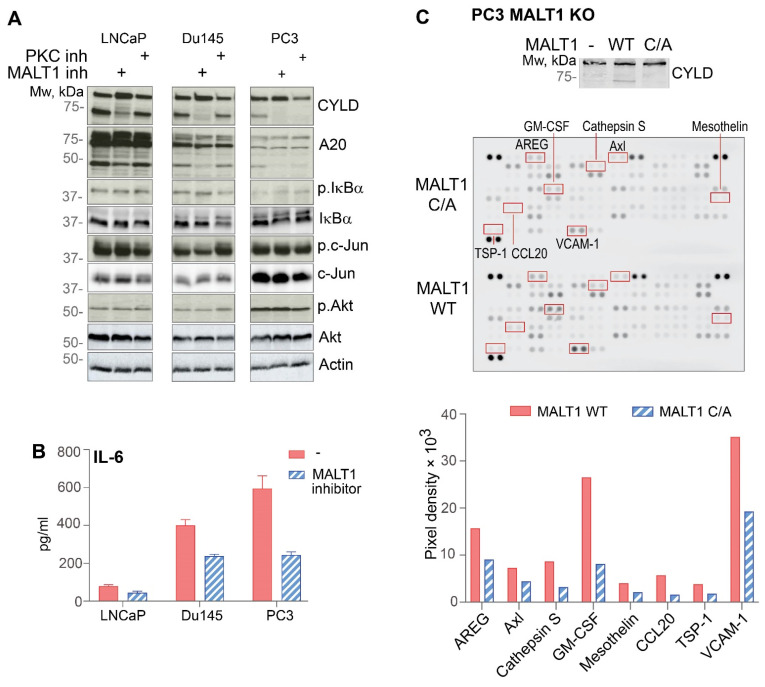
MALT1 proteolytic activity is not essential for constitutive NF-κB, JNK and Akt signalling in PCa cells, but contributes to the expression of cancer-related proteins. (**A**) PCa cell lines were treated with MALT1 (1 µM) or PKC inhibitor (10 µM) for 48 h (+ indicates each type of inhibitors applied to each cell line). CYLD and A20 processing, as well as c-Jun, IκBα, and Akt phosphorylation, were analysed by immunoblotting using the corresponding phospho-specific antibodies. (**B**) PCa cell lines were pre-treated with MALT1 inhibitor (2 µM) for 48 h, after which the media were refreshed with fresh inhibitor and analyzed for IL-6 expression in the supernatant by ELISA after additional 48 h. (**C**) PC3 *MALT1* KO cells were reconstituted with either wild type MALT1 (WT) or catalytically inactive MALT1 (C/A), as indicated. CYLD cleavage was visualised by western blot of the corresponding cell extracts. Cells were treated with Brefeldin A. After 6 h, expression of 84 human cancer-related proteins was analyzed via a multiplex antibody array. Quantification was conducted using ImageJ 1.53 software [45] and is shown in the bottom panel for a selected number of proteins (marked with red boxes). Results (**A**,**B**) are representative of three independent experiments. Error bars (**B**) represent means ± SEs.

Formation of the CARD14–BCL10–MALT1 complex induces activation of NF-κB and the MAP kinases p38 and JNK, which in turn induces the expression of several proinflammatory mediators (Figure 4A) [6]. NF-κB and JNK signalling have been shown to promote survival, proliferation, and invasion of PCa [5,41]. Therefore, we investigated the activation status of these pathways in PCa cells (LNCaP, PC3, and DU145) by analyzing IκBα and c-Jun phosphorylation, respectively. All three PCa cell lines showed constitutive IκBα and c-Jun phosphorylation (Figure 5A), indicative of constitutive CBM activity. We also observed constitutive Akt phosphorylation, which can mediate a number of downstream effects including mTOR activation, another key pathway in PCa [42]. Inhibition of MALT1 proteolytic activity by treatment of PCa cells with the MLT-827 had no effect on IκBα, c-Jun, or Akt phosphorylation again underscoring the dominant role of the scaffold function of MALT1 in CBM-mediated NF-κB and JNK signalling in PCa cells. Interestingly, treatment with a broad spectrum inhibitor of protein kinase C (PKC) enzymes, which are known upstream activators of CARD14 family proteins [43], not only failed to inhibit NF-κB/JNK/Akt phosphorylation, but also MALT1-mediated CYLD and A20 cleavage (Figure 5A), suggesting that constitutive CBM activation in PCa cells is mediated by a PKC-independent mechanism.

While the scaffold function of MALT1 is required for NF-κB activation and gene expression, MALT1 proteolytic activity mainly controls gene expression at the posttranscriptional level by regulating mRNA stability of specific genes, including *IL-6*, in an NF-κB-independent manner (Figure 4A) [40]. IL-6 is a cytokine that plays an important role in cancer progression [44], and treatments that inhibit IL-6 signalling are expected to have a beneficial effect in lymphoid malignancies, as well as in patients with solid tumours, including PCa. Therefore, we measured secretion of IL-6 by PCa cell lines in the presence or absence of MALT1 proteolytic activity. DU145 and PC3 cells were found to secrete high levels of IL-6, while LNCaP cells produced only minor amounts of this cytokine (Figure 5B). IL-6 secretion was significantly inhibited in DU145 and PC3 cells treated with MLT-827, suggesting that the proteolytic activity of MALT1 regulates IL-6 expression in these cells (Figure 5B). Tsui and colleagues previously suggested that knockdown of MALT1 in PC3 cells results in lower expression of IL-6 which correlated with reduced tumorigenesis in a xenograft mouse model [20]. Therefore, we further focused on PC3 cells to screen for cancer-related proteins that are regulated by the proteolytic activity of MALT1. To this end, we used *MALT1* knockout PC3 cells that were reconstituted with either wild type or catalytically inactive MALT1. MALT1 proteolytic function in reconstituted cells was verified by analysing CYLD cleavage (Figure 5C). While no CYLD cleavage could be observed in MALT1-deficient PC3 cells or cells reconstituted with the catalytically inactive mutant, wild type MALT1 restored CYLD cleavage in PC3 cells. We next used Human Oncology Array to compare 84 cancer-related proteins in PC3 cells expressing either wild type or catalytically inactive MALT1 (Figure 5C). This revealed that expression of several cancer-associated proteins, including GM-CSF, CCL20 and VCAM1, was strongly dependent on MALT1 catalytic activity in PC3 cells. Collectively, our results demonstrate that although CARD14-induced proteolytic activity of MALT1 does not regulate survival of LNCaP cells, constitutive activation of MALT1 contributes to the production of several cancer-related proteins, highlighting the potential of pharmacological inhibition of MALT1 as a novel therapeutic strategy for PCa.

## 4. Discussion

In the present study, we reported a novel functional role for CARD14 signaling in driving PCa cell survival. We showed that high expression of *CARD14* correlates with reduced patient survival, invasiveness, and recurrence in human PCa patients. Survival of LNCaP PCa cells was shown to be dependent on CARD14 expression and MALT1 scaffold activity. Moreover, we demonstrated constitutive MALT1 proteolytic activity in several PCa cell lines, which regulates the expression of several cancer-related genes.

Multiple cancers are driven by genetic or epigenetic alterations that dysregulate intracellular signalling pathways controlling cell proliferation and migratory capacity, cell survival, metabolism, genetic instability, and induction of angiogenesis. Anticancer drugs targeting such dysregulated signalling pathways in tumour cells are therefore of high interest, but the redundancy of pathways that control cell proliferation and survival presents a huge challenge. Moreover, many signalling pathways are shared by multiple cell types, leading to an increased risk of side effects on healthy cells. This is well illustrated by the NF-κB signalling pathway, which regulates the expression of several genes associated with the aforementioned hallmarks of cancer. However, NF-κB signalling also steers normal immune responses in multiple cell types; thus, the clinical development of NF-κB inhibitors as anti-cancer drugs has failed because of severe side effects. Overcoming these challenges requires the identification and detailed characterisation of more cell type-specific signalling molecules and mechanisms. In this context, our observation that high CARD14 expression in PCa patients correlates with more aggressive PCa, together with the finding that CARD14 expression is essential for survival of LNCaP PCa cells, reveal CARD14 as a novel anti-cancer target in PCa. Importantly, the rather specific expression of CARD14 in a subset of epithelial cells, together with the absence of a major phenotype in CARD14-deficient mice [46], suggests that side effects of specific CARD14 targeting may be quite limited. Until recently, intracellular adapter proteins such as CARD14 have long been considered undruggable, due to the absence of any enzymatic activity or active site. However in recent years, novel therapeutics against non-enzymatic targets has become more achievable with the development of PROteolysis TArgeting Chimera (PROTAC) inhibitors, bifunctional compounds capable of inducing target protein ubiquitination and proteasomal degradation [47]. Several PROTAC drugs are now under clinical development in cancer, and the field is rapidly growing. The future development of CARD14 PROTACs and the proof-of-concept of their use as therapeutics against certain prostate cancer types may have a high impact in the cancer field.

CARD14 signalling is mediated by MALT1, which has dual activities by acting as a scaffold which activates downstream NF-κB signalling, as well as by exerting proteolytic activity that regulates specific gene expression at the posttranscriptional level. Our data revealed that, similar to CARD14, MALT1 expression is also essential for LNCaP cell survival. Therefore, MALT1 targeting PROTACs may have potential for PCa treatment, although its ubiquitous expression may pose a significant risk for side effects. Alternatively, inhibition of CARD14 signalling can also be achieved by targeting MALT1 proteolytic activity using small molecule inhibitors. Importantly, although we showed that MALT1 enzymatic activity is not required for PCa cell survival, it does contribute to PCa tumour cell expression of several proteins that play key roles in PCa development and progression. Small molecule MALT1 inhibitors have already shown promising results in a number of preclinical cancer models for ABC-DLBCL, in which MALT1 is also constitutively activated [48]. However, several studies, including work from our group, have recently shown that long-term inhibition of MALT1 proteolytic activity is associated with systemic autoimmunity due to defective regulatory T cell function and increased effector T cell function, raising concerns about the safety of MALT1 inhibition [49,50,51,52]. Therefore, because of its more specific expression in certain epithelial cells, direct targeting of CARD14 (instead of MALT1) may be a more preferred approach in PCa treatment.

The mechanism of CBM complex activation in PCa cells remains to be elucidated. Activation of CARD-CC family members usually requires phosphorylation by specific PKCs [43]. However, we showed that PKC inhibitors failed to block CBM activation in PCa cells. It is possible that high expression of CARD14 in LNCaP cells (or possibly CARD10 in PC3 and DU145) is sufficient to overcome the autoinhibition of these proteins. In line with this, we previously showed that overexpression of CARD14 or CARD10, but not CARD11, is sufficient to initiate CBM signalling in HEK293T cells [43]. Alternatively, gain-of-function mutations of CARD14 are known to circumvent the need for upstream activation and initiate downstream signal transduction [6]. In this regard, it will be interesting to investigate the activity of CARD14 mutants that has been described in lethal castration-resistant PCa [26].

An important limitation of our current study is that the results presented here were obtained with PCa cell lines, failing to reproduce the complex tumour microenvironment. However, others have previously shown that xenograft tumour development was significantly attenuated when tumours were derived from PCa cells lacking MALT1 expression in a mouse model, supporting the role for CBM signalling in tumour formation in a complex microenvironment [19,20]. Further in vivo studies are expected to provide a better mechanistic understanding of the CARD14–BCL10–MALT1 axis in PCa and possibly even other carcinomas, eventually opening new therapeutic opportunities for targeting CARD14-mediated signalling in PCa and beyond.

## Data Availability

Publicly available datasets were analyzed in this study, as referred to in Materials and Methods section. Other data available on request.

## Supplementary Materials

The following supporting information can be downloaded at: https://www.mdpi.com/article/10.3390/biomedicines10082008/s1, Table S1. DNA damage response (DDR) gene list assembled from an online catalogue of DDR genes from recently published resources.
